# FSP27 Promotes Lipid Droplet Clustering and Then Fusion to Regulate Triglyceride Accumulation

**DOI:** 10.1371/journal.pone.0028614

**Published:** 2011-12-14

**Authors:** Srikarthika Jambunathan, Jun Yin, Waheed Khan, Yoshikazu Tamori, Vishwajeet Puri

**Affiliations:** 1 Department of Medicine, Section of Endocrinology, Diabetes and Nutrition, Boston University School of Medicine, Boston, Massachusetts, United States of America; 2 Division of Diabetes, Metabolism and Endocrinology, Department of Internal Medicine, Kobe University Graduate School of Medicine, Kobe, Japan; State University of Rio de Janeiro, Biomedical Center, Institute of Biology, Brazil

## Abstract

Fat Specific Protein 27 (FSP27), a lipid droplet (LD) associated protein in adipocytes, regulates triglyceride (TG) storage. In the present study we demonstrate that FSP27 plays a key role in LD morphology to accumulate TGs. We show here that FSP27 promotes clustering of the LDs which is followed by their fusion into fewer and enlarged droplets. To map the domains of FSP27 responsible for these events, we generated GFP-fusion constructs of deletion mutants of FSP27. Microscopic analysis revealed that amino acids 173–220 of FSP27 are necessary and sufficient for both the targeting of FSP27 to LDs and the initial clustering of the droplets. Amino acids 120–140 are essential but not sufficient for LD enlargement, whereas amino acids 120–210 are necessary and sufficient for both clustering and fusion of LDs to form enlarged droplets. In addition, we found that FSP27-mediated enlargement of LDs, but not their clustering, is associated with triglyceride accumulation. These results suggest a model in which FSP27 facilitates LD clustering and then promotes their fusion to form enlarged droplets in two discrete, sequential steps, and a subsequent triglyceride accumulation.

## Introduction

Cellular lipid droplets (LDs) are dynamic organelles which regulate triglyceride (TG) stores in cells [1], [2], [3], [4]. LDs are composed of a core of neutral lipids surrounded by a phospholipid monolayer and associated proteins [5], [6], [7]. Of the LD-associated proteins, the best-characterized proteins are members of the PAT family, also called the perilipin (Plin) family, of proteins [3], [4], [8] which function in the regulation of lipolysis. We and others identified another LD associated protein that is highly expressed in adipocytes, Fat Specific Protein 27 (FSP27), and plays a unique role in LD dynamics. Accumulating evidence indicates that FSP27 plays a role in TG accumulation and LD size in adipocytes [9], [10], [11] and liver [12]. Depletion of FSP27 in cultured adipocytes causes LD fragmentation and an increase in lipolysis, whereas its expression in non-adipose cells increases LD size and TG levels [9], [10]. A non-sense mutation in the C-terminus of CIDEC (human ortholog of FSP27) in humans also results in the accumulation of multiple, small LD's in white adipocytes *in vivo* [13]. These results suggest the role of FSP27 in regulating LD morphology, but the mechanism(s) by which FSP27 regulates LD morphology is not known.

In our preliminary studies, and by closely examining published images [9], [10], [14], we noted that overexpression of FSP27 in COS-7 and 293T cells initially leads to the clustering of small lipid LDs before larger ones appear. We therefore hypothesized that the FSP27-mediated increase in LD size is not a simple process but involves multiple steps and is regulated by specific domains of FSP27. Here we show that FSP27 promotes LD clustering which is then followed by the formation of fewer and enlarged LDs. Our data also reveal that LD enlargement but not clustering causes TG accumulation. A recent study identified that LD localization of FSP27 requires amino acids 174–192 of its C-domain [15]. In this study Liu et al. showed that amino acids 174–192 are required but not sufficient for LD localization of FSP27. Thus, our objective in the current study was to define the domains of FSP27 that are required for LD targeting and for LD enlargement. While confirming the importance of the C-terminus of FSP27 in LD localization, our results show that the amino acids 173–220 of FSP27 play a role in targeting of FSP27 to LDs and their clustering. We have demonstrated that amino acids 120–140 are essential but not sufficient for LD enlargement and that amino acids 120–210 are sufficient for both clustering and fusion of LDs to form enlarged droplets. Taken together, our data show that FSP27 regulates LD morphology and TG accumulation in cells by first clustering the LDs and then fusing them to form fewer and enlarged LDs. Thus, these results indicate a direct regulatory role of FSP27 in LD dynamics.

## Results

### FSP27-GFP expression causes clustering of LDs

**Figure 1:**
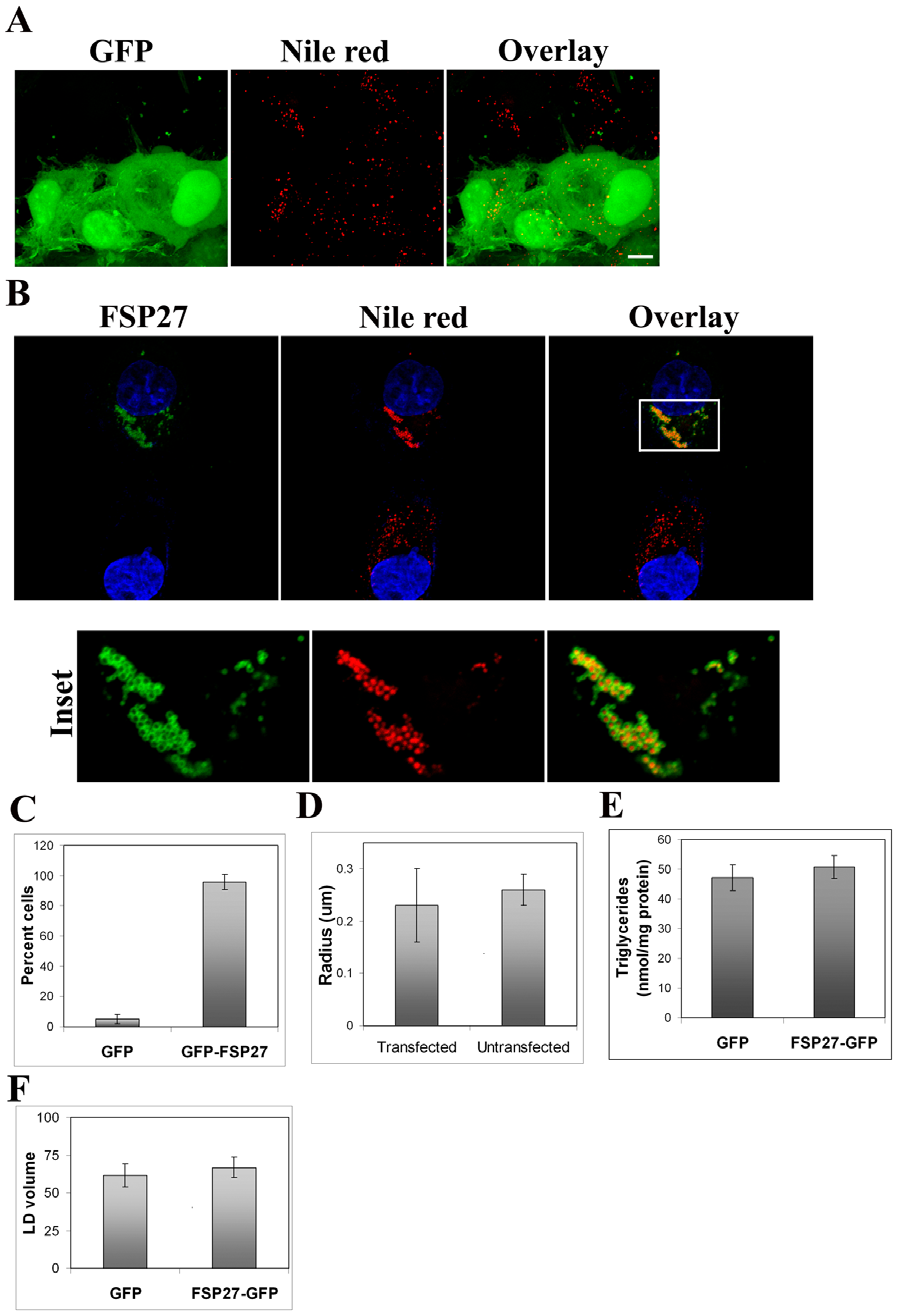
FSP27-GFP expression causes clustering of LDs in COS-7 cells. **A**, COS-7 cells were transfected with GFP vector alone. Left panel shows GFP expression in cells after 16 hr of transfection. The LDs stained with Nile red are shown in red color in the middle panel. Bar, 10 µm. **B**, COS-7 cells transfected with FSP27-GFP for 16 hr. Nucleus was stained with DAPI. Left panel shows cell expressing FSP27-GFP (top) and non-expressing cell (bottom) in the same fied of the microscope. The middle panel shows LDs stained with Nile red. A 2 µm Z-slice of the transfected cell (in the inset of top right panel) is enlarged in the lower panels to show a clear representation of FSS7-GFP distribution around the clustered droplets (also see **Figure S1**). Bar, 10 µm. **C**, percent of GFP or FSP27-GFP expressing COS-7 cells which showed clustered LDs. Results are an average of at least three independent experiments ± standard deviation. About 25 cells were counted in each individual experiment (p<0.0001). **D**, morphometric analysis was performed to measure the radius of LDs in FSP27-GFP transfected and untransfected COS-7 cells. Results are an average of at least 25 cells from three independent experiments ± standard deviation. **E**, Biochemical quantification of total triglycerides in COS-7 cells after 16 hr of transfection with GFP and FSP27-GFP cDNA. Data is from three independent experiments, error bars show standard deviations (p = 0.17). **F**, total LD volume in cells transfected with GFP vector alone and FSP27-GFP. The bars indicate the volume in µm^3^ ± standard error, (*, p = 0.3).

In order to define the role of FSP27 in LD morphology we carried out our initial studies in COS-7 cells because these cells do not have endogenous FSP27 or other adipogenic proteins such as PPARγ and perilipin (PLIN1) [3], [16], and these cells have been widely used as a model system to study the signaling, transport or interactions of various adipocyte specific proteins [17], [18], [19], [20], [21], [22], [23], [24], or to study the function of various LD proteins and lipases [27], [28], [29]. GFP vector alone had no effect on the homogeneous LD distribution in COS-7 cells (Fig. 1A), whereas overnight (16 hr) FSP27-GFP expression caused aggregation or clustering of LDs (Fig. 1B). Furthermore, as shown in previous studies [9], [10], [14], [30], FSP27 was associated with LDs and showed a ring-like pattern around the LDs in 2-dimensional imaging. As shown in Figure 1C, about 85% of FSP27 expressing cells showed clustered LDs. The enlarged view of LDs in FSP27-GFP-expressing and non-expressing cells from Fig. 1B is shown in Figure S1. Morphometric analysis of LDs in FSP27-GFP transfected and untransfected cells confirmed that clustering of droplets had no significant effect on the average size of LDs (Fig. 1D).

To ensure that the LD clustering was not an artifact of the GFP fused to FSP27, we performed the experiment with HA-tagged FSP27. A similar intracellular LD distribution pattern was observed in FSP27-HA expressing COS-7 cells (Figure S2), indicating that FSP27 localization around the LDs and FSP27-mediated clustering of LDs is not influenced by GFP or HA tags. Also, note that GFP, HA, Flag or DsRed tags fused to FSP27 or other CIDE proteins do not alter their respective properties of LD targeting and/or enlargement [10], [14], [31], [32].

Finally, we studied the effect of FSP27 on cellular TG levels. Biochemical analysis was performed to quantify total cellular TG levels after 16 hr of transfecting GFP vector alone or FSP27-GFP in COS-7 cells. Transfection efficiency of GFP vector alone and FSP27-GFP in COS-7 cells was 95% and 50% respectively. As shown in Fig. 1E, clustering of LDs had no significant effect on total TG levels. Since the transfection efficiency of FSP27-GFP was low in COS-7 cells, therefore in order to further confirm that there was no effect of clustering on TG levels we measured total LD volume as an indirect measure of TG levels. As shown in Fig. 1F, there was no significant change in the total LD volume per cell in cells transfected with GFP vector alone or FSP27-GFP.

In order to confirm that the phenomenon of LD clustering is not specific to COS-7 cells we expressed FSP27-GFP in brown and 3T3-L1 preadipocytes. After 16 hr of transfecting FSP27-GFP clustering of LDs was observed in 3T3-L1 preadipocytes (Figure S3) and cultured brown preadipocytes (Figure S4) which expressed FSP27-GFP. To further confirm that FSP27-GFP could cluster the LDs in adipocytes, we expressed FSP27-GFP in 3T3-L1 adipocytes at day 3 of their differentiation. As shown in Figure S5, FSP27-GFP expression caused clustering of LDs in differentiating 3T3-L1 adipocytes with FSP27 forming a ring-like pattern around the LDs.

### Amino acids 173–220 are necessary and sufficient for both targeting of FSP27 to LDs and clustering of LDs

**Figure 2:**
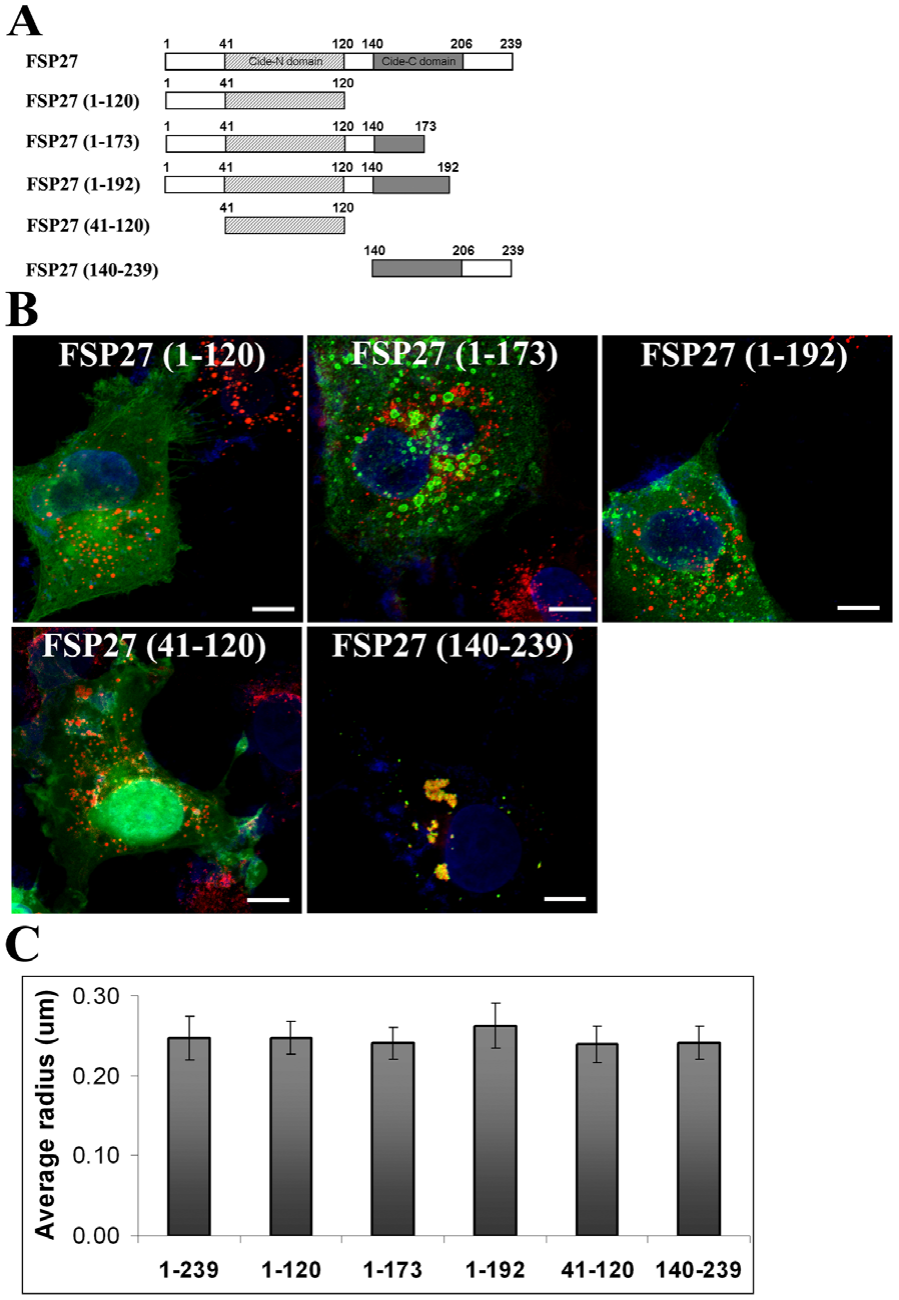
Carboxy-terminus of FSP27 is necessary for LD targeting and clustering. **A**, deletion mutations of FSP27. GFP fusion constructs of these deletion mutations were prepared to identify the LD targeting and clustering regions of FSP27. **B**, expression of GFP fusion constructs of FSP27 deletion mutants in COS-7 cells. The images show the distribution of mutants (green) after 16 hr of transfection. LDs were labeled with Nile red (red) and nucleus was labeled with DAPI (blue). **C**, morphometric analysis was performed on microscopic images to measure the radius of LDs. The results shown are an average of mean radius of LDs in at least 10 cells from three different experiments in each condition. The error bars show standard deviation.

To identify the functional domains of FSP27 that are required for its LD targeting and clustering of the droplets, we prepared truncation mutants of FSP27 fused to GFP and studied their effect on FSP27 localization and LD arrangement. The domains chosen were based on our recent studies on the sequence homology of FSP27 to the PAT-family protein PLIN1 [33] and a study by Liu et al. [15] where they showed that amino acids 174–192 are required but not sufficient for LD localization of FSP27. The truncation constructs (Fig. 2A) generated include the amino terminal 1–120 amino acids (FSP27 (1–220)), Cide-N domain of amino acids 41–120 (FSP27 (41–120)), amino acids 1–173 (FSP27 (1–173)), amino acids 1–192 (FSP27 (1–192)) and the carboxy terminal 140–239 (FSP27 (140–239)). FSP27 (1–120), FSP27 (41–120), FSP27 (1–173) and FSP27 (1–192) showed diffused cytoplasmic and/or nuclear staining with LDs scattered throughout the cytoplasm (Fig. 2B). In contrast, FSP27 (140–239) was localized to LDs and caused their clustering (Fig. 2B). There was no difference in the size of LDs in any of the above cases (Fig. 2C).

**Figure 3:**
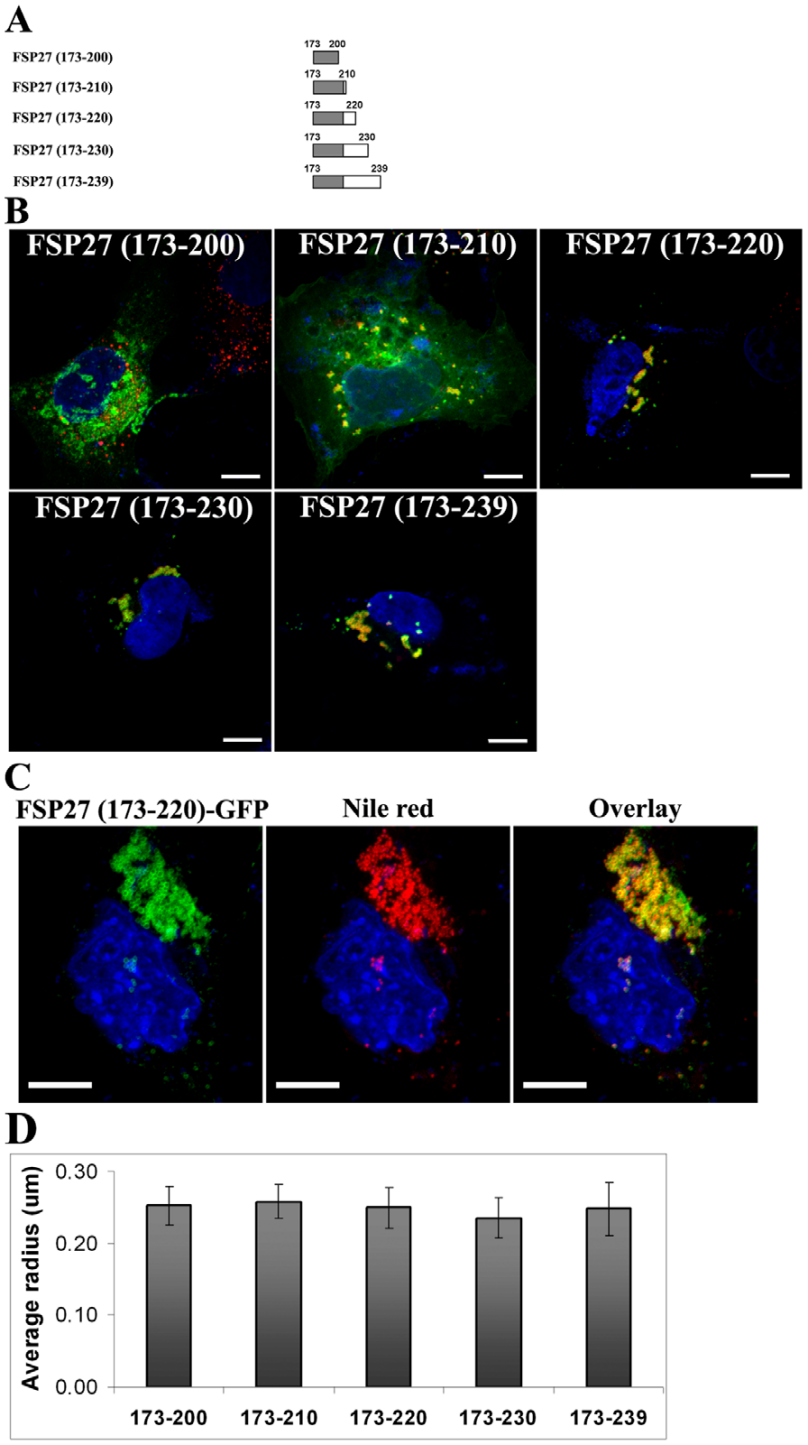
Amino acids 173–220 are necessary and sufficient for localization of FSP27 to LDs and their clustering. **A**, deletion mutants in the C-terminus of FSP27 which were fused with GFP to identify the minimum essential domain of FSP27 required for its targeting to LDs and cluster them. **B**, expression of GFP fusion constructs of FSP27 deletion mutants in COS-7 cells. The images show the distribution of mutants (green) after 16 hr of transfection. LDs were labeled with Nile red (red) and nucleus was labeled with DAPI (blue). **C**, amino acids 173–220 of FSP27 are necessary and sufficient for targeting to the LDs and clustering them. Left panel shows FSP27 (173–220)-GFP expression after 16 hr of transfection, middle panel shows the LDs labeled with Nile red. Bar 10 µm. **D**, morphometric analysis was performed on microscopic images to measure the radius of LDs. The results shown are an average of mean radius of LDs in at least 10 cells from three different experiments in each condition. The error bars show standard deviation.

The data above revealed that the carboxy-terminus of FSP27 is important for its localization to LDs and their clustering. We next attempted to identify the smallest amino acid sequence in the carboxy-terminus of FSP27 responsible for its LD targeting and clustering. Since Liu et al. [15] have shown that 174–192 are essential for LD localization of FSP27 [15], we prepared GFP constructs of amino acids 173–200 (FSP27 (173–200)), 173–210 (FSP27 (173–220)), 173–220 (FSP27 (173–220)) and 173–239 (FSP27 (173–239)) (Fig. 3A). FSP27 (173–200) expression in COS-7 cells showed diffused staining with no affect on LD distribution, FSP27 (173–210) showed partial localization to LDs and caused some clustering of LDs, whereas FSP27 (173–220), FSP27 (173–230) and FSP27 (173–239) were completely localized to LDs and clustered the LDs (Fig. 3B). FSP27 (173–220) was the smallest sequence which had a localization pattern similar to full length FSP27-GFP, with ring-like labeling surrounding LDs (Figs. 3B and 3C). Also, FSP27 (173–220) clustered the LDs the same as full length FSP27-GFP. The expression of all of the above constructs had no effect on the size of the LDs (Fig. 3D).

### The clustering of LDs is followed by fewer and enlarged LDs

**Figure 4:**
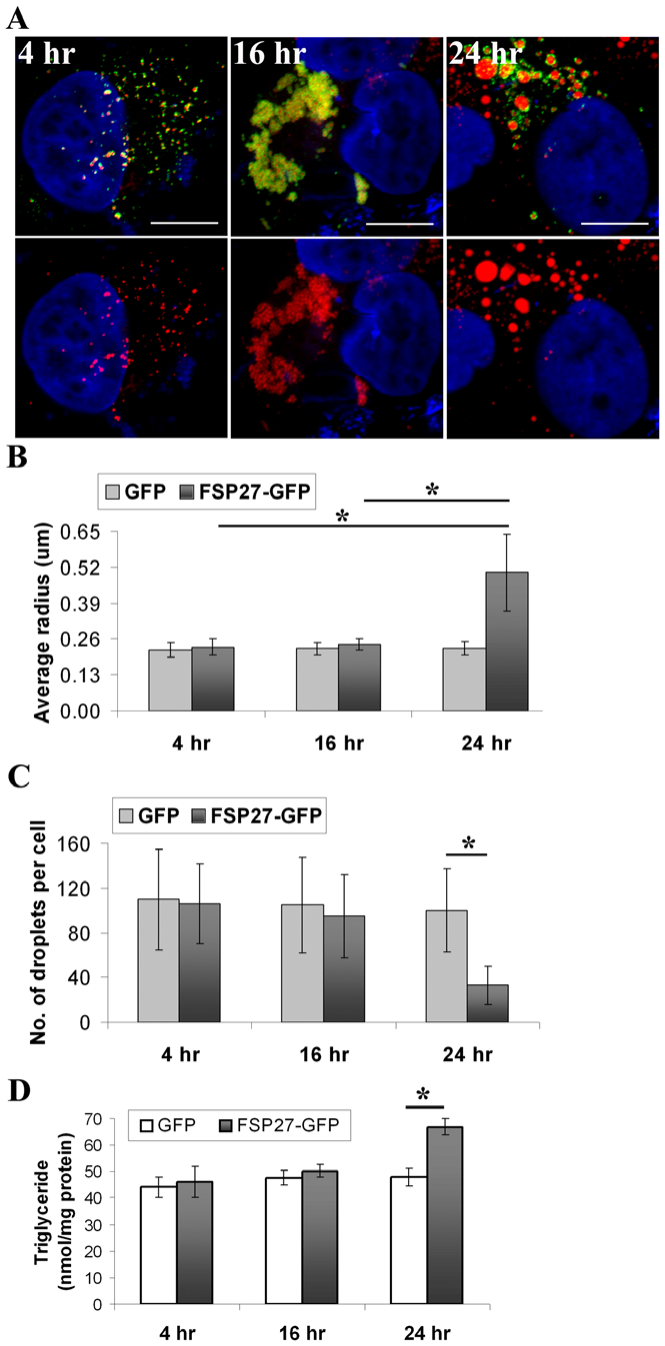
Clustering of LDs is followed by their enlargement in FSP27-GFP expressing COS-7 cells. A, time course of FSP27-GFP expression in COS-7 cells. COS-7 cells were transfected with FSP27-GFP. After 4 hr, 16 hr and 24 hr of transfection, the cells were fixed, labeled with Nile red and analyzed under confocal microscope. After 4 hr of FSP27 expression almost all the LDs were associated with FSP27 (left panel), at 16 hr the LDs in about 85% of FSP27-GFP expressing cells were clustered (middle panel) and at 24 hr after FSP27 transfection about 80–85% of the cells had enlarged LDs (right panel). Bar 10 µm. **B**, Morphometric analysis was performed on microscopic images to measure the average radius of LDs. The results are from at least 10 cells in each condition form three independent experiments. The error bars show standard deviation, (*, p<0.001). **C**, Quantification of LDs per cell was performed in GFP and FSP27-GFP expressing cells at different time points. The results are an average of LDs per cell. LDs in at least 10 cells were counted in each condition from three independent experiments, (*, p<0.001). **D**, biochemical quantification of total TGs in COS-7 cells at different time points after transfection with GFP and FSP27-GFP. Data is from three independent experiments, error bars show standard deviation, (*, p<0.001).

In order to further study the consequence of clustered LDs on LD morphology, we performed a time course to follow the effect of FSP27-GFP expression on LD morphology. After 4 hr of transfecting FSP27-GFP 92±4% of the cells with green fluorescence had LDs associated with FSP27-GFP (Fig. 4A). The LDs were not clustered at this time point; however, almost all the LDs in cells had FSP27-GFP associated with them. At the 16 hr time point, 85±3% of FSP27-GFP-expressing cells showed clustering, though the extent of LD clustering varied among cells. Further incubation of these cells for about 6–8 hr resulted in fewer and enlarged LDs with FSP27 still associated with them. 84±5% of FSP27-GFP-expressing cells had enlarged LDs. The micrographs were generated by stacking 3 µm Z-sections of confocal images. The individual slices were examined carefully to rule out the possibility of overlap of LDs in different focal planes which might resemble enlarged LDs. The average radius of enlarged LDs was increased 2–3 fold (Fig. 4B). Quantification of number of droplets per cell confirmed that at the 24 hr time point, FSP27-GFP expressing cells had significantly reduced number of LDs as compared to the GFP-expressing cells (Fig. 4C). These results show that after 24 hr of FSP27-GFP transfection, cells had fewer but larger LDs.

Previously it has been found that FSP27 regulates TG storage in cells [9], [10], [11]; therefore, in order to find out if FSP27-mediated clustering and/or enlargement were associated with TG accumulation in the cells, we performed biochemical analysis of TG content of the cells at different time points after GFP or FSP27-GFP transfection. As shown in Figure 4D, there was no change in TG content at the 4 hr and 16 hr time points, whereas at 24 hr about 40% increase in total TG content was observed. These results show that FSP27-mediated clustering has no effect on TG accumulation, whereas the enlargement of LDs is accompanied by TG accumulation.

### Amino acids 120–210 are necessary and sufficient for FSP27-mediated LD enlargement

**Figure 5:**
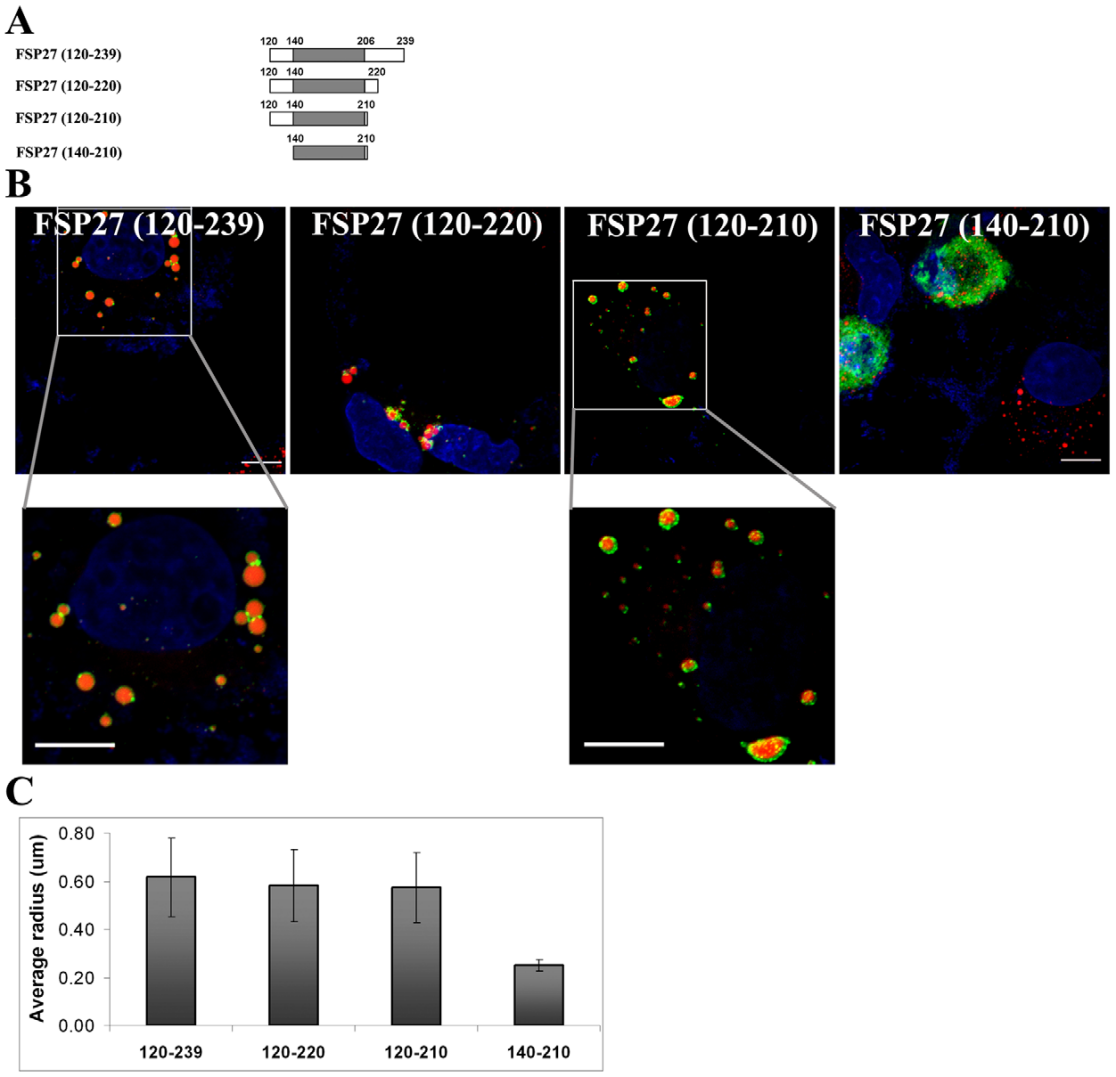
Amino acids 120–210 are necessary and sufficient for enlargement of LDs. **A**, deletion mutants of FSP27 which were fused with GFP to identify the minimum essential domain of FSP27 associated with LD enlargement. **B**, expression of GFP fusion constructs of FSP27 deletion mutants in COS-7 cells. The images show the distribution of mutants (green) after 16 hr of transfection. LDs were labeled with Nile red (red) and nucleus was labeled with DAPI (blue). Bar 10 µm. **C**, morphometric analysis was performed on microscopic images to measure the radius of LDs. The results shown are an average of mean radius of LDs in at least 10 cells from three different experiments in each condition. The error bars show standard deviation.

The amino acid sequence 173–220 was responsible for localization of FSP27 to LDs and their clustering but its expression had no affect on the size of LDs. We hypothesized that a separate domain of FSP27 is responsible for LD enlargement. Amino acid sequence 140–239 had no effect on the size of LDs (Fig. 2C), therefore we added the amino acids ranging from 120–140 to the sequence 140–239. We picked this 20 amino acid region because adding these amino acids to 140–239 would complete the carboxy-terminus of FSP27. Also, this is the region between the CIDE-N and CIDE-C domains of FSP27. A GFP-fusion construct with amino acids 120–239 (FSP27 (120–239)) (Fig. 5A) was expressed in cells. As shown in Fig. 5B, after 16 hr of transfection FSP27 (120–239) caused fewer and enlarged LDs. Interestingly, the enlargement of LDs was also observed even after 8 hr of expressing FSP27 (120–239) (data not shown). These results suggest that the 20 amino acids from 120–140 are essential for FSP27-mediated LD enlargement.

In order to find out the shortest sequence of FSP27 that is sufficient for LD enlargement, we prepared GFP-fusion constructs of amino acids ranging from 120–220 (FSP27 (120–220)) and 120–210 (FSP27 (120–210)). As shown in Figs. 5B and 5C, expression of both FSP27 (120–220) and FSP27 (120–210) led to fewer and enlarged LDs in cells. Expression of amino acids 120–200 had no significant effect on LD size (data not shown). These results indicate that amino acids 120–210 are sufficient for FSP27-mediated LD enlargement.

**Figure 6:**
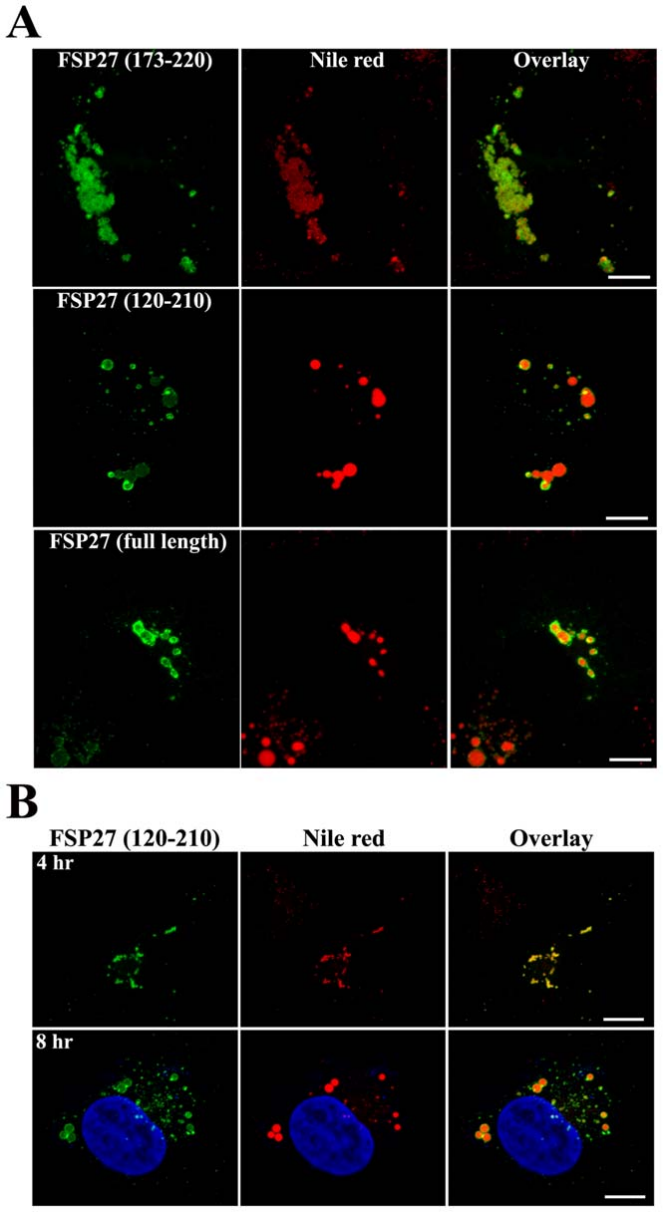
FSP27 (120–210) rapidly clusters and enlarges the LDs. **A**, LD morphology after 24 hr of transfecting FSP27 (173–220), FSP27 (120–210), and full length FSP27 in COS-7 cells. LDs were stained with Nile red. Bar 10 µm. **B**, COS-7 cells were transfected with FSP27 (120–210) and analyzed after 4 hr and 8 hr of transfection. LDs were stained with Nile red. Bar 10 µm.

In our studies FSP27 (173–220) clustered the LDs after 16 hr of transfection which was similar to full length FSP27 (Fig. 3B, C). But in the case of full length FSP27 the clustering of LDs was followed by their enlargement in the next 8 hrs. Therefore, in order to confirm that amino acids 173–220 were associated with only clustering of LDs but not their enlargement, we incubated the COS cells for 24 hrs after transfecting them with FSP27 (173–220). As shown in Figure 6A, FSP (173–220) expression after 24 hr of transfection showed only clustering of LDs. On the other hand full length FSP27 showed the formation of larger LDs under the same conditions.

Interestingly, the LD enlargement by amino acids 120–210 occurred in a shorter time (16 hr) compared to the full length FSP27 (24 hr). This indicates that this region of FSP27 is a core domain in promoting the enlargement of LDs. However, it was not clear yet whether the FSP27 (120–210) induced LD enlargement followed their clustering or enlargement in this case skipped the clustering of LDs. Therefore, we next performed the experiment with FSP27 (120–210) for shorter time points than 16 hr. As shown in Fig. 6B, 4 hr after transfecting FSP27 (120–210) the LDs were clustered. This clustering of LDs was followed by the formation of fewer and enlarged LDs in the next 4 hr. FSP27 (120–210) was also localized to the small droplets scattered throughout the cytoplasm. These LDs could be newly formed droplets associated with FSP27 (120–210) which were not yet clustered and enlarged. Taken together, the above results show that amino acids 120–210 are sufficient for both clustering and enlargement of LDs.

### FSP27 might play a role in LD coalescence to form enlarged LDs

**Figure 7:**
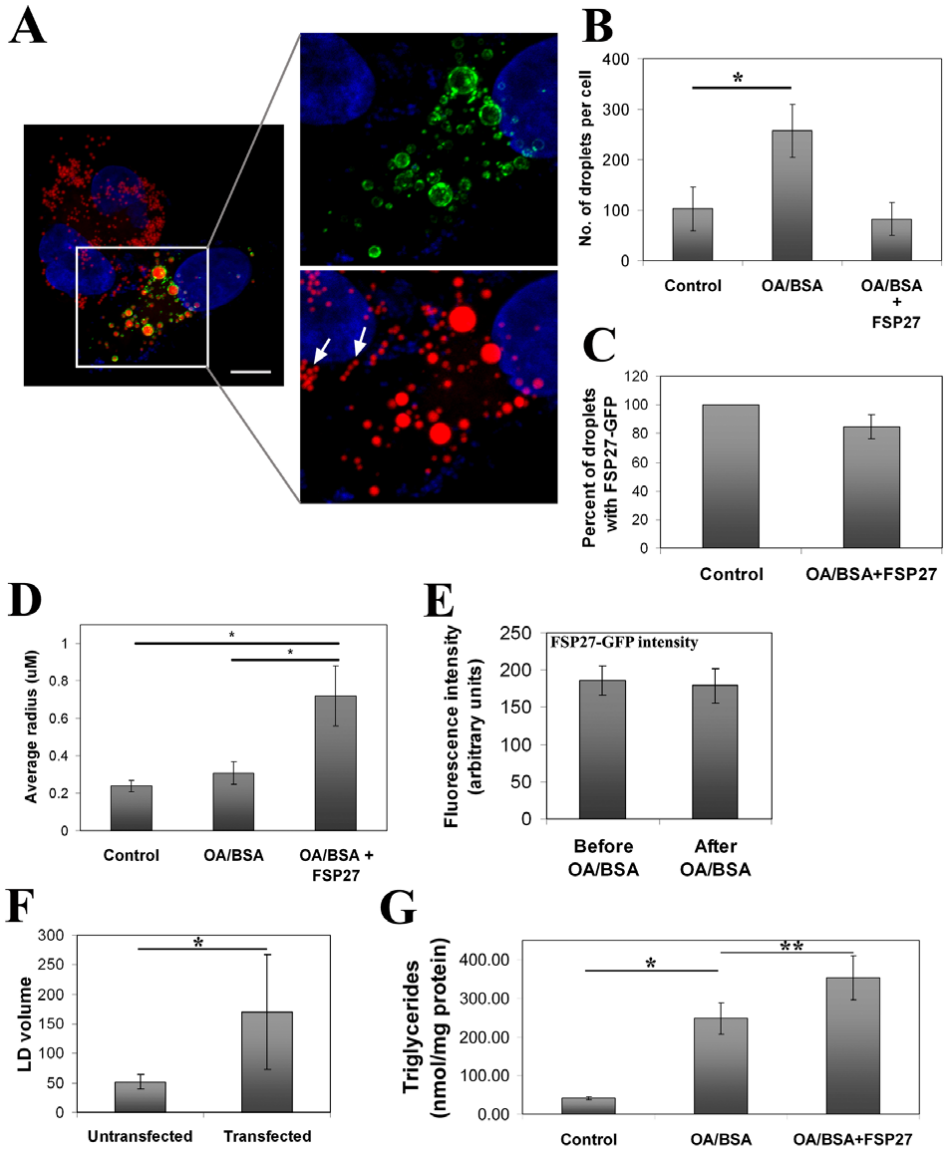
FSP27 redistributes to newly formed LDs and might promote coalescence of droplets to form enlarged LDs. **A**, COS-7 cells were transfected with FSP27-GFP, 4 hr after transfection 70 µM cycloheximide was added for 1 hr to inhibit protein synthesis. The cells were then fed with 60 µM OA/BSA for the formation of new LDs overnight during which 70 µM cycloheximide was also present. After overnight incubation the cells were washed, incubated for another 1 hr in the absence of OA/BSA and presence of cycloheximide, fixed and stained with Nile red. Note that the FSP27-GFP expressing cells have fewer but enlarged LDs and FSP27-GFP was associated with most of the droplets independent of their size. In contrast, the neighboring cells that do not express FSP27-GFP, are filled with large numbers of small LDs and all of these droplets are of similar size, also, in spite of close packing of the droplets (shown by arrows in the right bottom image) they do not show any sign of coalescence. **B**, quantification of number of droplets per cell under conditions mentioned above. In control, the cells were fed with OA/BSA alone. (*, p<0.0001). **C**, percent of droplets associated with FSP27-GFP after overnight OA/BSA treatment of COS-7 cells. **D**, average radius of the lipid droplets. In control, the cells were fed with BSA alone (*, p<0.0001). **E**, fluorescence intensity of FSP27-GFP before and after OA/BSA feeding in the presence of cycloheximide under the conditions mentioned in panel A. **F**, total LD volume in untransfected and FSP27-GFP transfected cells fed with OA/BSA. The bars indicate the volume in µm^3^ ± standard deviation. (*, p<0.002). **G**, biochemical analysis of TG content in COS-7 cells. The cells transfected with GFP had 5.5-fold increase in TGs when fed with OA/BSA (*, p<0.0001). The cells transfected with FSP27-GFP and fed with OA/BSA showed further increase in TG content (**, p<0.005). In B–F, the cells were treated with cycloheximide and fed with OA/BSA in the exact same conditions mentioned in panel A.

The FSP27-mediated clustering of LDs was followed by fewer and enlarged droplets, suggesting that the enlargement of clustered LDs was due to their coalescence. In order to test that FSP27 might be promoting the coalescence of LDs to form enlarged LDs, we transfected cells with FSP27-GFP and incubated for 4 hr to allow FSP27 to be expressed. The cells were then incubated with 70 µM cycloheximide for 1 hr to inhibit FSP27 synthesis. Further incubation of these cells was carried out in the presence of 60 µM OA/BSA and 70 µM cycloheximide. After overnight incubation OA/BSA was removed and the cells were incubated for another 1 hr in the presence of cycloheximide to give some time to the newly formed LDs to mature. The cells were washed, fixed and stained with Nile red. Microcopic images showed that FSP27 was associated with most of the droplets in OA/BSA fed cells (Figure 7A). Under our experimental conditions, the untransfected cells were filled with a large number of small LDs formed in the presence of exogenous OA/BSA, and all of these droplets were of similar size. Interestingly, in spite of close packing of the droplets (shown by arrows) in the cells not expressing FSP27-GFP, the LDs did not show any sign of coalescence (Figure 7A). As shown in Fig. 7B, the total number of droplets in OA/BSA fed cells, which did not express FSP27-GFP, increased almost 2.5-fold as compared to the cells that were treated with BSA alone (control). In contrast OA/BSA fed cells that expressed FSP27 (OA/BSA+FSP27) had fewer but larger droplets compared to the cells that did not express FSP27-GFP. Also, about 85% of the droplets in OA/BSA+FSP27 cells had FSP27 associated with them (Figure 7C). Morphometric analysis showed significantly enlarged LDs in FSP27-GFP expressing cells (Figure 7D). Almost all the cells that expressed FSP27-GFP had fewer and enlarged LDs.

Since most of the droplets in OA/BSA+FSP27 cells were associated with FSP27, this suggests that it could be existing FSP27 protein which was redistributed to the newly formed droplets. To confirm whether the existing protein was redistributed to LDs, the intensity of GFP fluorescence was measured before and after OA/BSA feeding in the presence of cycloheximide. There was no marked difference in total fluorescence intensity of FSP27-GFP per cell before and after OA/BSA treatment in the presence of cycloheximide (Fig. 7E), indicating the redistribution of the existing FSP27 on the LDs.

The total volume of LDs was increased about 3-fold in FSP27 expressing cells compared to the neighboring untransfected cells (Figure 7F), which indicates higher TG levels in FSP27 expressing cells. To further confirm that TGs were higher in FSP27 expressing cells, we performed biochemical analysis of total TGs in the cells transfected with FSP27-GFP or GFP vector alone under the above mentioned conditions. FSP27 increased TG content of the cells by 45% (Figure 7G).

The above data suggested that FSP27 promotes coalescence of LDs to form enlarged LDs. Thus, we next attempted to study the distribution of FSP27 in the above studies at shorter time points. The cells were transfected with FSP27-GFP; 4 hr later they were incubated with 70 µM cycloheximide for 1 hr and then fed with OA/BSA in the presence of cycloheximide. After 3 hr the cells were washed, fixed and labeled with Nile red for confocal microscopy. As shown in Figure S6, FSP27-GFP was associated with most of the droplets and appeared to connect the droplets. Many droplets also gave the appearance that they were undergoing coalescence. Figure S7 shows another cell where almost all the LDs in the FSP27 expressing cell were clustered and gave an appearance as if they were undergoing coalescence with FSP27-GFP forming a mesh around all the LDs. No such phenotype was observed in the neighboring non-FSP27 expressing cell (Figure S7).

## Discussion

**Figure 8:**
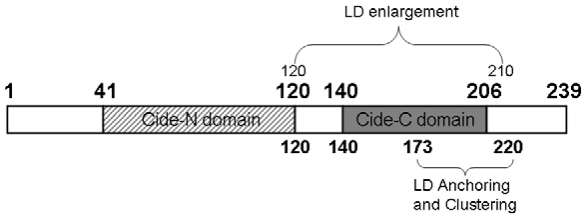
Schematic representation of FSP27 and its functional domains. Two domains of FSP27 have been reported in literature, CIDE-N and CIDE-C. The CIDE-N and CIDE-C domain ranges from amino acids 41–120 and 140–206 respectively [48]. The N-terminus of FSP27 is from amino acids 1–120 and the C-terminus is from amino acids 121–239. We found that FSP27-mediated enlargement of LDs consists of two independent steps, clustering followed by fusion of LDs. Amino acids 172–210 are necessary and sufficient for FSP27-mediated clustering of LDs. The clustering of LDs has no effect on their size and cellular TG levels. The LD clustering is followed by their enlargement. Amino acids 120–210 are sufficient for clustering and enlargement of LDs. Interestingly, FSP27-mediated enlargement of LDs is accompanied with increased TG levels in the cells. These results highlight the important role of FSP27 in LD morphology and TG accumulation.

The mechanisms by which cells accumulate triglycerides in distinct organelles called LDs, and especially how the size and number of these LDs are regulated, remain poorly understood. Primary adipocytes, which express a number of LD proteins, including PAT proteins and FSP27, accumulate lipid in one main LD while other cell types remain multilocular [34], [35]. In fact, in all cells, there is a dynamic regulation of LD morphology. The current work demonstrates that FSP27-mediated enlargement of LDs is not a simple process, but rather consists of two independent steps. Initially FSP27 mediates a rearrangement of small LDs to form clusters, and then it promotes the formation of fewer and enlarged droplets by fusing the clustered droplets. Amino acids 173–220 in the C-terminus of FSP27 play a role in localization of FSP27 to LDs and also clusters the LDs. Furthermore, we found that the amino acids 120–210, which overlap most of 173–220, are necessary and sufficient for both clustering and fusing the droplets to form enlarged droplets (Figure 8). These effects were observed in COS-7 cells, which lack key lipases and other LD proteins that are present in adipocytes [3], suggesting the unique importance of FSP27 in the regulation of TG accumulation and LD morphology.

Our data advance the knowledge of the specific domains of FSP27 that mediate its LD targeting, the clustering of newly formed LDs, and their apparent coalescence and enlargement. The data are consistent with a recent study by Liu et al. [15] which documented the importance of the C-terminus of FSP27 in LD anchoring. They found that amino acids 174–192 are required but not sufficient for LD localization of FSP27. In a collaborative study, we have previously shown that a non-sense mutation in the C-terminus of human FSP27 (CIDEC) results in a truncation in the protein which causes multiple small LD's in white adipocytes of a human subject [13]. Consistent with these studies, we found that amino acids 1–120, which belong to N-terminus of FSP27 had no role in localization of FSP27 to LDs. We further identified amino acids 173–220 in the C-terminus of FSP27 as sufficient for both its localization to LDs and their clustering. Furthermore, we found that amino acids 120–210 are necessary and sufficient for both clustering and enlargement of LDs. In this domain, amino acids 120–140 had an important role in regulating LD enlargement. Without residues 120–140 the remaining portion of the FSP27, i.e. 140–239, could not enlarge the LDs but only cluster them (Fig. 2), suggesting that residues 120–140 play an essential role in the coalescence of the clustered LDs to form enlarged droplets. Taken together, these data suggest that amino acids 120–210 facilitates the fusion of LDs, and in this fusogenic domain of FSP27 the amino acid residues 120–140 might play an important role in LD fusion. The residues 120–140 connect CIDE-N and CIDE-C domains of FSP27 (Fig. 8). According to the secondary structure prediction using SWISS-MODEL [36], most of the residues in the 120–140 region form a coil in the protein structure. The 120–140 sequence is a mixture of acidic, basic and hydrophobic amino acid residues. Interestingly, the model did not predict any transmembrane helices in the FSP27 protein.

FSP27 (120–210)-mediated LD enlargement was preceded by clustering of droplets. LD size was increased by the FSP27 (120–210) mutant even in 8 hr. This time course is faster than that of full length FSP27. It indicates that the 120–210 amino acid region is a core domain in promoting the clustering and fusion of LDs. The two functional domains of FSP27, amino acids 173–220 and 120–210, overlap significantly. Interestingly, the domain of FSP27, 120–210 includes all the amino acids from the clustering domain of 173–220 except for the residues 210–220. In fact, amino acids 173–210 did show clustering of LDs along with both LD and cytosolic localization of the mutant protein (Fig. 3B). Possibly, 120–210 being a core domain in promoting LD enlargement caused more rapid coalescence of clustered droplets. Moreover, there was no difference in the size of the enlarged LDs by both FSP27 (120–210) and FSP27 (120–220) mutants (Fig. 5C), which shows that amino acids 120–210 are sufficient to cause LD enlargement. Nonetheless, our study has identified that amino acids 120–210 of FSP27 protein play a role in LD fusion to form enlarged LDs. It seems logical that for the coalescence of LDs to form larger droplets, the droplets have to come together in contact with each other. Indeed, our study demonstrates that the clustering is a prerequisite for FSP27-mediated enlargement of LDs and the two steps mediated by FSP27, i.e. clustering and enlargement of LDs, are discrete and sequential. Overall our data show that FSP27 causes fusion of LDs to form enlarged droplets and that the amino acid domain 120–210 plays an important role in this function of FSP27.

FSP27 and another CIDE-family protein, Cidea, share about 60% sequence similarity in their amino acids [33].The C-terminus of Cidea was shown to be critical for LD anchoring and TG accumulation in cells [32]. In this study Christianson et al. showed that the C-terminal domain of Cidea directs LD targeting, clustering, and TG accumulation, whereas the N-terminal domain is required for Cidea-mediated development of enlarged LDs. FSP27 has a total of 239 amino acids; therefore the amino acids from 120 onwards belong to the C-terminal. Our study shows that amino acids 120–210 are necessary and sufficient for LD enlargement. Overall our studies indicate that the C-terminal of FSP27 plays a role in anchoring FSP27 to LDs and regulating LD morphology. Interestingly, based on alignment of the amino acid residues 120–140 of FSP27 with full length Cidea, there is no sequence similarity in amino acids. It indicates that the two proteins, Cidea and FSP27, play a redundant role in LD enlargement but domains consisting of different amino acids are involved in this function. This hypothesis at least is in consonance with the difference between Cidea and FSP27, i.e. N-terminal of Cidea [32] but C-terminal of FSP27 are critical for the development of enlarged LDs.

Our data show that FSP27 through its amino acids 120–210 causes fusion of LDs but do not provide a mechanism. It is possible that FSP27 causes clustering of LDs and they coalesce due to lipid-lipid hydrophobic interactions. This is most likely not the case, as the incubation of non-FSP27-GFP cells with OA/BSA resulted in numerous LDs which were in contact with each other, but enlarged LDs were not formed in the absence of FSP27 (Figure 7A); we did not observe any sign of coalescence or fusion of the LDs of these cells. In contrast, the FSP27 expressing cells displayed significantly larger LDs. Another point of interest with respect to the current study concerns the distorted shape of LDs which were undergoing coalescence (Figures S6 and S7). The distortion of LDs was not observed in the cells not expressing FSP27 (Figure S7); which rules out the possibility that it is an artifact due to fixing of the cells before microscopy. In fact, we used paraformaldehyde for fixing the cells, which is shown to be a method of choice to study LDs microscopically [37]. The distortion in coalescing LDs indicates that the FSP27-mediated coalescence of LDs is a complex process which might require a rearrangement of the LD surface prior to the complete fusion of LDs. Indeed a recent study by Murphy et al. [38] showed that LD fusion occurs in two stages in 3T3-L1 adipocytes: an initial rapid fusion of the surfaces of LDs to form a continuous membrane, followed by a slower merging of the contents and the reformation of a single round LD. The role of FSP27 in these processes needs to be determined experimentally.

Our studies using COS-7 cells, which do not express adipocyte-specific LD proteins such as perilipin, indicate that FSP27 might not require the adipocyte-specific proteins to regulate the clustering or enlargement of LDs. On the other hand, FSP27 may work in concert with other proteins like SNARE proteins which are ubiquitously expressed and have been shown to mediate fusion between cytosolic LDs [39], [40]. Interestingly, FSP27 was observed reaching from one droplet to another (Figure S6). A recent study has indicated that FSP27 has a capability to form homodimers and heterodimers [15]. Therefore, it could be that FSP27 forms homodimers that help FSP27 to extend from one droplet to another to bring the LDs together. Future studies will address whether the truncation mutants of FSP27 have any effect on homodimeric interactions of FSP27. Although the distribution pattern of FSP27-GFP, i.e. reaching from one droplet to another, was observed in multiple cells, the possibility is that it could be incidental. The extended FSP27-GFP signal could be due to association with the small LDs that failed to get stained with nile red, giving an appearance of FSP27 extending to the other LDs. It could also be due to other cellular organelles, like endoplasmic reticulum, at the same focal plane where FSP27-GFP might be localized. Nonetheless, the distribution of FSP27 which shows that it reaches from one droplet to another and appears to bind them together (shown in inset 1 and 2 of Figure S6) could explain its role in rearrangement of the droplets to bring them together. Further studies are required to explain this distribution pattern of FSP27.

Our data suggest that FSP27 regulates LD morphology changes in multiple steps to form large LDs in adipocytes. Previous studies have shown that FSP27 depletion in adipocytes causes fragmentation of LDs and increased lipolysis [9], [10]. Also, FSP27 deficiency blocked the maturation of LDs during adipogenesis in 3T3-L1 adipocytes; that is, in FSP27 shRNA expressing cells, small LDs were distributed throughout the cytoplasm whereas adipocytes with normal FSP27 expression had large LDs (data not shown), a typical phenotype of differentiated adipocytes. In fact, a recent study by Li et al [41] has shown small LDs and less TG accumulation in CIDEC deficient human adipocytes. In this study they found that CIDEC-deficient human adipocytes have low perilipin and higher PPARγ mRNA levels and therefore proposed that CIDEC might be involved in the differentiation of adipocytes. On the contrary, Keller et al [10] previously suggested that FSP27 knockdown does not impair adipogenesis in cultured adipocytes. Furthermore, FSP27 knockout mice develop white adipose tissue with multilocular adipocytes [14], [34], [42] which indicates that FSP27 could be involved in maintaining LD morphology during adipogenesis and may not affect the differentiation of adipocytes, which is a highly regulated process. Nonetheless, these studies suggest that FSP27 is associated with TG accumulation and formation of large LDs in cultured adipocytes.

In summary, our data suggest that FSP27-mediated clustering and enlargement of LDs are two distinct and sequential steps. The clustering of LDs via FSP27 had no effect on LD size, but it was followed by fewer and enlarged LDs. Amino acids 120–210 are necessary and sufficient for both clustering and fusion of LDs to form enlarged droplets. Furthermore, our results show that enlargement of LDs, but not their clustering, is associated with increased TG levels in the cells. This FSP27-mediated enlargement of LDs is not due simply to an increase in TG synthesis since the role of FSP27 in TG synthesis has already been ruled out [12]. This could be due to the fact that the enlarged LDs have decreased lipolysis due to decreased surface area/volume. Indeed, our previous study reveals that FSP27 negatively regulates lipolysis and promotes triglyceride accumulation [9]. Overall, our study helps to gain a better understanding of the steps involved in FSP27-mediated LD morphology and TG storage. Further studies are required to understand the mechanism of FSP27-mediated clustering and fusion of LDs in adipocytes as it applies to the efficiency and capacity of adipocytes to store TG in adipocytes, a phenomenon which is associated with the pathophysiology of diseases such as Type 2 diabetes and obesity [43], [44], [45], [46], [47].

## Materials and Methods

### Materials

Nocodazole (Sigma Aldrich, MO), Latrunculin A (Sigma Aldrich, MO), Mitotracker (Invitrogen, CA), Nile Red (Sigma Aldrich, MO), HCS LipidTOX-Deep Red (Invitrogen, CA), Cycloheximide (Sigma Aldrich, MO), OA/BSA (Sigma Aldrich, MO). COS-7 cells were purchased from ATCC. pEGFPC1 vector was purchased from Clontech.

### DNA Plasmids and constructs

The FSP27 cDNA was purchased from Open Biosystems. PCR was performed to generate DNA encoding a full-length or truncated product by using a 5′-linker containing a BglII restriction site and a 3′-linker containing an EcoRI site. After cutting with the restriction enzymes (BglII and EcoRI), the purified PCR fragments were cloned into pEGFP-C1 vector (Clontech). All constructs were verified by full sequencing of inserts (Molecular Genetics Core facility, Boston University School of Medicine, Boston, MA).

### Cell Culture and transfections

Cells were cultured in DMEM supplemented with 10% FBS, 50 µg/ml streptomycin, and 50 units/ml penicillin. For experiments performed in COS-7 cells and preadipocytes, 5×10^5^ cells were electroporated with 10 µg of plasmid DNA. The electroporation was performed using a Bio-Rad GenePulser Xcell in a 0.2-cm cuvette at a setting of 110V, 20 milliseconds, and single pulse. Immediately after electroporation, the cells were reseeded and incubated for 16 hrs before being fixed with paraformaldehyde and stained with Nile Red for confocal microscopy [37].

### Fluorescence microscopy

Microscopy was performed using a Zeiss LSM 710-Live Duo scan (Carl Zeiss, Oberkochen, Germany) with a ×100 oil immersion objective. Cells plated on glass coverslips were washed twice with PBS and fixed with 4% paraformaldehyde post-transfection. Lipid droplets were stained with 0.5 µg/ml of Nile Red or HCS LipidTOX-Deep Red stain by incubating cells for 15 minutes. Images were processed using Metamorph imaging software, version 6.1 (Universal Imaging, Downingtown, PA).

### Lipid droplet staining

Cells were washed with PBS, fixed in 4% Formaldehyde for 20 minutes and quenched with 0.1 M glycine. Cells were then incubated with 0.5 µg/ml of Nile Red or HCS LipidTOX-Deep Red stain for 30 minutes. Cells were then washed with PBS and the cover slips were mounted on glass slides with VectaShield (Vector Labs, CA) mounting media.

### Morphometric analysis on lipid droplets

The morphometric analysis on lipid droplets was performed on confocal images by using Metamorph version 7.1. After subtracting the background, the cells were outlined and threshold for Nile red intensity. Each droplet was identified as a discrete object using object classifiers in metamorph program. Only the droplets that were identified individually by the program were chosen for analysis. Pixel-to-micrometer ratio was assigned, and the program measured the radius and volume (equivalent sphere volume) of the Nile red-stained lipid droplets in cubic micrometers. In each condition, 10–25 cells were measured for total volume of droplets per cell.

### Triglyceride Determination

Cells were transfected as described above and at 16 hour post-transfection, wells were lysed with cell lysis buffer (CellSignal), sonicated, and quantified for TG using the TG Determination Kit (Sigma) according to the manufacturer's instructions.

## Supporting Information

Figure S1 **The middle panel of** **Figure 1B** **from the manuscript is enlarged to show the distribution and morphology of LDs.** The FSP27-GFP expressing and non-expressing cells are shown in the same field at the same magnification.
(TIF)

Figure S2 **FSP27-HA expression causes clustering of LDs in COS-7 cells.** Cos-7 cells were transfected with cDNA of FSP27 fused to HA tag. After 16 hr of transfection the cells were fixed with paraformaldehyde and stained with HA-antibodies. Bar, 10 µm.
(TIF)

Figure S3 **FSP27-GFP expression causes clustering of LDs in 3T3-L1 preadipocyes.** The 3T3-L1 preadipocytes were transfected with FSP27-GFP cDNA and incubated with 20 µM OA/BSA for 16 hr. LDs were stained with Nile red.
(TIF)

Figure S4 **FSP27-GFP expression in brown preadipocytes causes clustering of LDs.** Brown preadipocytes after 16 hr of transfection with GFP (top panels) and FSP27-GFP (bottom panels). LDs were stained with Nile red (middle panel).
(TIF)

Figure S5 **FSP27-GFP causes clustering of LDs in differentiating 3T3-L1 adipocytes.** FSP27-GFP was expressed in differentiating 3T3-L1 adipocytes on day 3. 16 hr after transfection the cells were fixed and observed under confocal microscope. Left panel shows the distribution of LDs where all the confocal Z-sections of the cell were stacked to form one single image. The right hand side panel shows a very thin slice of 2 µm showing clustered LDs. Bar, 10 µm.
(TIF)

Figure S6 **FSP27-GFP distribution during LD enlargement.** FSP27 binds various LDs together by extending from one LD to another. The cells were transfected with FSP27-GFP, 4 hr after transfection cycloheximide was added for 1 hr and then the cells were fed with OA/BSA for 3 hr in the presence of cycloheximide. LDs were stained with Nile red. Inset shows that FSP27 is extended from one droplet to another (Arrows) and it is concentrated on most of the points from where it is extended. Bar, 10 µm.
(TIF)

Figure S7 **FSP27 might facilitate fusion of LDs.** The cells were transfected with FSP27-GFP, 4 hr after transfection cycloheximide was added for 1 hr and then the cells were fed with OA/BSA overnight in the presence of cycloheximide. LDs were stained with Nile red. Note that FSP27 forms a kind of mesh around the droplets which gave an appearance as if they were coalescing. The untransfected cell within the same field has number of droplets distributed throughout the cytoplasm. Bar 10 µm.
(TIF)

## References

[1] Beckman M (2006). Cell biology. Great balls of fat.. Science.

[2] Farese RV, Walther TC (2009). Lipid droplets finally get a little R-E-S-P-E-C-T.. Cell.

[3] Brasaemle DL (2007). Thematic review series: adipocyte biology. The perilipin family of structural lipid droplet proteins: stabilization of lipid droplets and control of lipolysis.. J Lipid Res.

[4] Ducharme NA, Bickel PE (2008). Lipid droplets in lipogenesis and lipolysis.. Endocrinology.

[5] Wolins NE, Brasaemle DL, Bickel PE (2006). A proposed model of fat packaging by exchangeable lipid droplet proteins.. FEBS Lett.

[6] Martin S, Parton RG (2006). Lipid droplets: a unified view of a dynamic organelle.. Nat Rev Mol Cell Biol.

[7] Fujimoto T, Parton RG (2011). Not just fat: the structure and function of the lipid droplet.. Cold Spring Harb Perspect Biol.

[8] Kimmel AR, Brasaemle DL, McAndrews-Hill M, Sztalryd C, Londos C (2010). Adoption of PERILIPIN as a unifying nomenclature for the mammalian PAT-family of intracellular lipid storage droplet proteins.. J Lipid Res.

[9] Puri V, Konda S, Ranjit S, Aouadi M, Chawla A (2007). Fat-specific protein 27, a novel lipid droplet protein that enhances triglyceride storage.. J Biol Chem.

[10] Keller P, Petrie JT, De Rose P, Gerin I, Wright WS (2008). Fat-specific protein 27 regulates storage of triacylglycerol.. J Biol Chem.

[11] Brasaemle DL, Dolios G, Shapiro L, Wang R (2004). Proteomic analysis of proteins associated with lipid droplets of basal and lipolytically stimulated 3T3-L1 adipocytes.. J Biol Chem.

[12] Matsusue K, Kusakabe T, Noguchi T, Takiguchi S, Suzuki T (2008). Hepatic steatosis in leptin-deficient mice is promoted by the PPARgamma target gene Fsp27.. Cell Metab.

[13] Rubio-Cabezas O, Puri V, Murano I, Saudek V, Semple RK (2009). Partial lipodystrophy and insulin resistant diabetes in a patient with a homozygous nonsense mutation in CIDEC.. EMBO Mol Med.

[14] Nishino N, Tamori Y, Tateya S, Kawaguchi T, Shibakusa T (2008). FSP27 contributes to efficient energy storage in murine white adipocytes by promoting the formation of unilocular lipid droplets.. J Clin Invest.

[15] Liu K, Zhou S, Kim JY, Tillison K, Majors D (2009). Functional analysis of FSP27 protein regions for lipid droplet localization, caspase-dependent apoptosis, and dimerization with CIDEA.. Am J Physiol Endocrinol Metab.

[16] Fang X, Dillon JS, Hu S, Harmon SD, Yao J (2007). 20-carboxy-arachidonic acid is a dual activator of peroxisome proliferator-activated receptors alpha and gamma.. Prostaglandins Other Lipid Mediat.

[17] Berger J, Bailey P, Biswas C, Cullinan CA, Doebber TW (1996). Thiazolidinediones produce a conformational change in peroxisomal proliferator-activated receptor-gamma: binding and activation correlate with antidiabetic actions in db/db mice.. Endocrinology.

[18] Castillo G, Brun RP, Rosenfield JK, Hauser S, Park CW (1999). An adipogenic cofactor bound by the differentiation domain of PPARgamma.. EMBO J.

[19] Gronke S, Mildner A, Fellert S, Tennagels N, Petry S (2005). Brummer lipase is an evolutionary conserved fat storage regulator in Drosophila.. Cell Metab.

[20] Hasegawa T, Matsuzaki T, Tajika Y, Ablimit A, Suzuki T (2007). Differential localization of aquaporin-2 and glucose transporter 4 in polarized MDCK cells.. Histochem Cell Biol.

[21] Lu X, Murphy TC, Nanes MS, Hart CM (2010). PPAR{gamma} regulates hypoxia-induced Nox4 expression in human pulmonary artery smooth muscle cells through NF-{kappa}B.. Am J Physiol Lung Cell Mol Physiol.

[22] Martinez-Arca S, Lalioti VS, Sandoval IV (2000). Intracellular targeting and retention of the glucose transporter GLUT4 by the perinuclear storage compartment involves distinct carboxyl-tail motifs.. J Cell Sci.

[23] Wang Y, Sullivan S, Trujillo M, Lee MJ, Schneider SH (2003). Perilipin expression in human adipose tissues: effects of severe obesity, gender, and depot.. Obes Res.

[24] Yamazaki T, Zaal K, Hailey D, Presley J, Lippincott-Schwartz J (2002). Role of Grb2 in EGF-stimulated EGFR internalization.. J Cell Sci.

[25] Zhang DE, Hetherington CJ, Meyers S, Rhoades KL, Larson CJ (1996). CCAAT enhancer-binding protein (C/EBP) and AML1 (CBF alpha2) synergistically activate the macrophage colony-stimulating factor receptor promoter.. Mol Cell Biol.

[26] Villena JA, Roy S, Sarkadi-Nagy E, Kim KH, Sul HS (2004). Desnutrin, an adipocyte gene encoding a novel patatin domain-containing protein, is induced by fasting and glucocorticoids: ectopic expression of desnutrin increases triglyceride hydrolysis.. J Biol Chem.

[27] Wolins NE, Quaynor BK, Skinner JR, Tzekov A, Croce MA (2006). OXPAT/PAT-1 is a PPAR-induced lipid droplet protein that promotes fatty acid utilization.. Diabetes.

[28] Lass A, Zimmermann R, Haemmerle G, Riederer M, Schoiswohl G (2006). Adipose triglyceride lipase-mediated lipolysis of cellular fat stores is activated by CGI-58 and defective in Chanarin-Dorfman Syndrome.. Cell Metab.

[29] Granneman JG, Moore HP, Mottillo EP, Zhu Z, Zhou L (2011). Interactions of perilipin-5 (Plin5) with adipose triglyceride lipase.. J Biol Chem.

[30] Kim JY, Liu K, Zhou S, Tillison K, Wu Y (2008). Assessment of fat-specific protein 27 in the adipocyte lineage suggests a dual role for FSP27 in adipocyte metabolism and cell death.. Am J Physiol Endocrinol Metab.

[31] Ranjit S, Boutet E, Gandhi P, Prot M, Tamori Y (2011). Regulation of fat specific protein 27 by isoproterenol and TNF-alpha to control lipolysis in murine adipocytes.. J Lipid Res.

[32] Christianson JL, Boutet E, Puri V, Chawla A, Czech MP (2010). Identification of the lipid droplet targeting domain of the Cidea protein.. J Lipid Res.

[33] Puri V, Ranjit S, Konda S, Nicoloro SM, Straubhaar J (2008). Cidea is associated with lipid droplets and insulin sensitivity in humans.. Proc Natl Acad Sci U S A.

[34] Puri V, Czech MP (2008). Lipid droplets: FSP27 knockout enhances their sizzle.. J Clin Invest.

[35] Greenberg AS, Coleman RA, Kraemer FB, McManaman JL, Obin MS (2011). The role of lipid droplets in metabolic disease in rodents and humans.. J Clin Invest.

[36] Arnold K, Bordoli L, Kopp J, Schwede T (2006). The SWISS-MODEL workspace: a web-based environment for protein structure homology modelling.. Bioinformatics.

[37] DiDonato D, Brasaemle DL (2003). Fixation methods for the study of lipid droplets by immunofluorescence microscopy.. J Histochem Cytochem.

[38] Murphy S, Martin S, Parton RG (2010). Quantitative analysis of lipid droplet fusion: inefficient steady state fusion but rapid stimulation by chemical fusogens.. PLoS One.

[39] Bostrom P, Andersson L, Rutberg M, Perman J, Lidberg U (2007). SNARE proteins mediate fusion between cytosolic lipid droplets and are implicated in insulin sensitivity.. Nat Cell Biol.

[40] Bostrom P, Andersson L, Li L, Perkins R, Hojlund K (2009). The assembly of lipid droplets and its relation to cellular insulin sensitivity.. Biochem Soc Trans.

[41] Li F, Gu Y, Dong W, Li H, Zhang L (2010). Cell death-inducing DFF45-like effector, a lipid droplet-associated protein, might be involved in the differentiation of human adipocytes.. FEBS J.

[42] Toh SY, Gong J, Du G, Li JZ, Yang S (2008). Up-regulation of mitochondrial activity and acquirement of brown adipose tissue-like property in the white adipose tissue of fsp27 deficient mice.. PLoS One.

[43] Bays H, Mandarino L, DeFronzo RA (2004). Role of the adipocyte, free fatty acids, and ectopic fat in pathogenesis of type 2 diabetes mellitus: peroxisomal proliferator-activated receptor agonists provide a rational therapeutic approach.. J Clin Endocrinol Metab.

[44] Boden G, Shulman GI (2002). Free fatty acids in obesity and type 2 diabetes: defining their role in the development of insulin resistance and beta-cell dysfunction.. Eur J Clin Invest.

[45] Kashyap S, Belfort R, Gastaldelli A, Pratipanawatr T, Berria R (2003). A sustained increase in plasma free fatty acids impairs insulin secretion in nondiabetic subjects genetically predisposed to develop type 2 diabetes.. Diabetes.

[46] Paolisso G, Gambardella A, Amato L, Tortoriello R, D'Amore A (1995). Opposite effects of short- and long-term fatty acid infusion on insulin secretion in healthy subjects.. Diabetologia.

[47] Guilherme A, Virbasius JV, Puri V, Czech MP (2008). Adipocyte dysfunctions linking obesity to insulin resistance and type 2 diabetes.. Nat Rev Mol Cell Biol.

[48] Inohara N, Koseki T, Chen S, Wu X, Nunez G (1998). CIDE, a novel family of cell death activators with homology to the 45 kDa subunit of the DNA fragmentation factor.. Embo J.

