# Supplementary figures and images for: FSP27 Promotes Lipid Droplet Clustering and Then Fusion to Regulate Triglyceride Accumulation
Source: PLoS One. 2011 Dec 14;6(12):e28614. doi: 10.1371/journal.pone.0028614 (PMC3237475; doi:10.1371/journal.pone.0028614)

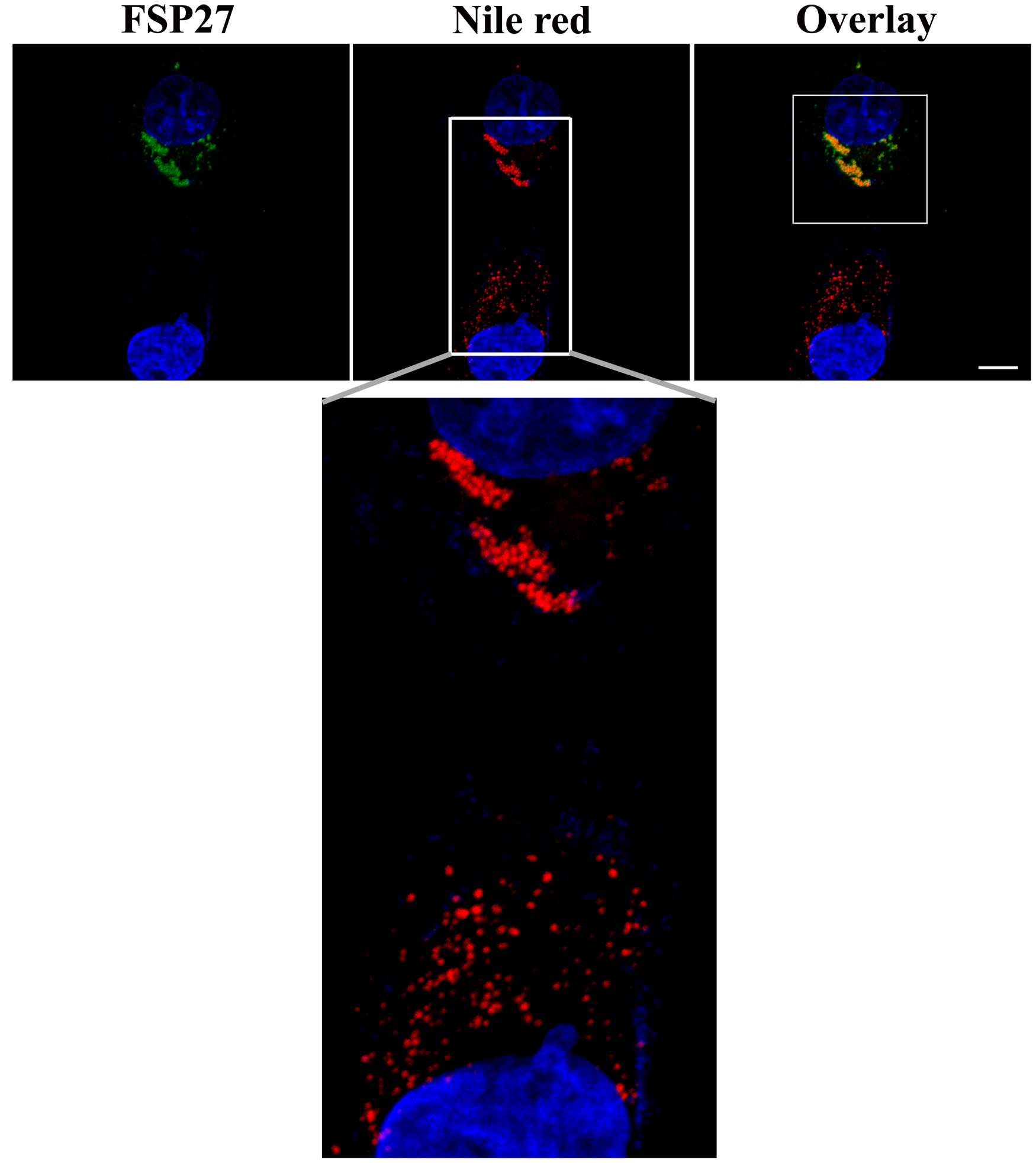

Supplement: Figure S1 — The middle panel of Figure 1B from the manuscript is enlarged to show the distribution and morphology of LDs. The FSP27-GFP expressing and non-expressing cells are shown in the same field at the same magnification. (TIF) [file pone.0028614.s001.tif]

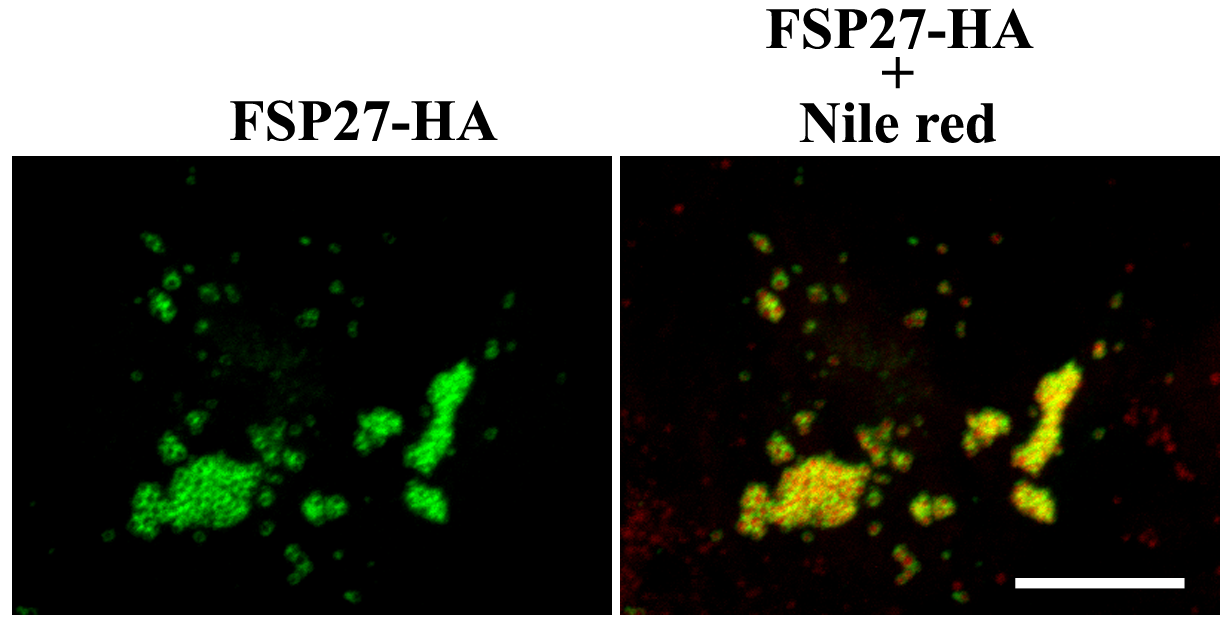

Supplement: Figure S2 — FSP27-HA expression causes clustering of LDs in COS-7 cells. Cos-7 cells were transfected with cDNA of FSP27 fused to HA tag. After 16 hr of transfection the cells were fixed with paraformaldehyde and stained with HA-antibodies. Bar, 10 µm. (TIF) [file pone.0028614.s002.tif]

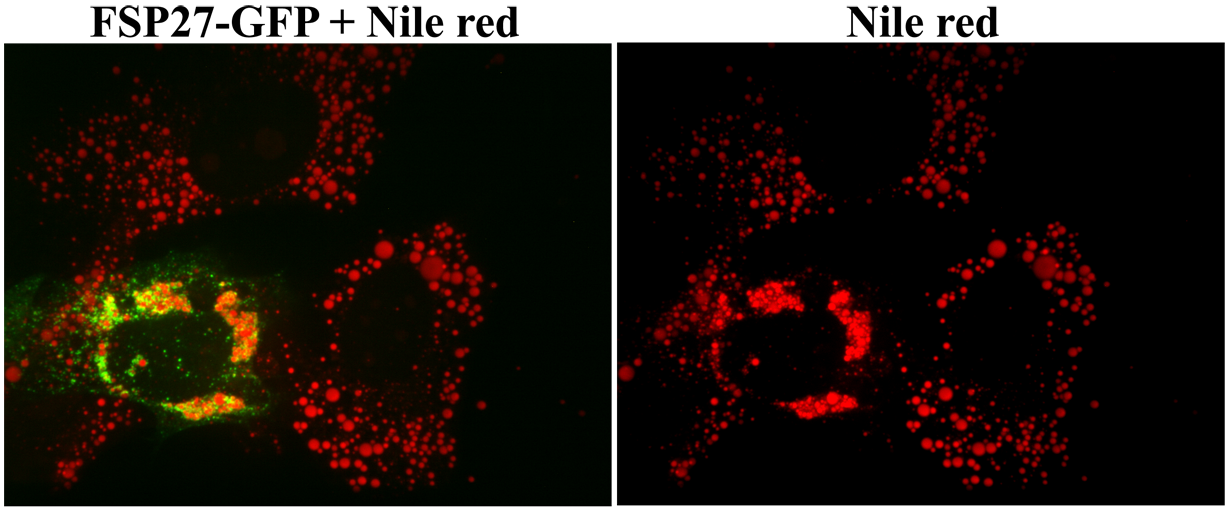

Supplement: Figure S3 — FSP27-GFP expression causes clustering of LDs in 3T3-L1 preadipocyes. The 3T3-L1 preadipocytes were transfected with FSP27-GFP cDNA and incubated with 20 µM OA/BSA for 16 hr. LDs were stained with Nile red. (TIF) [file pone.0028614.s003.tif]

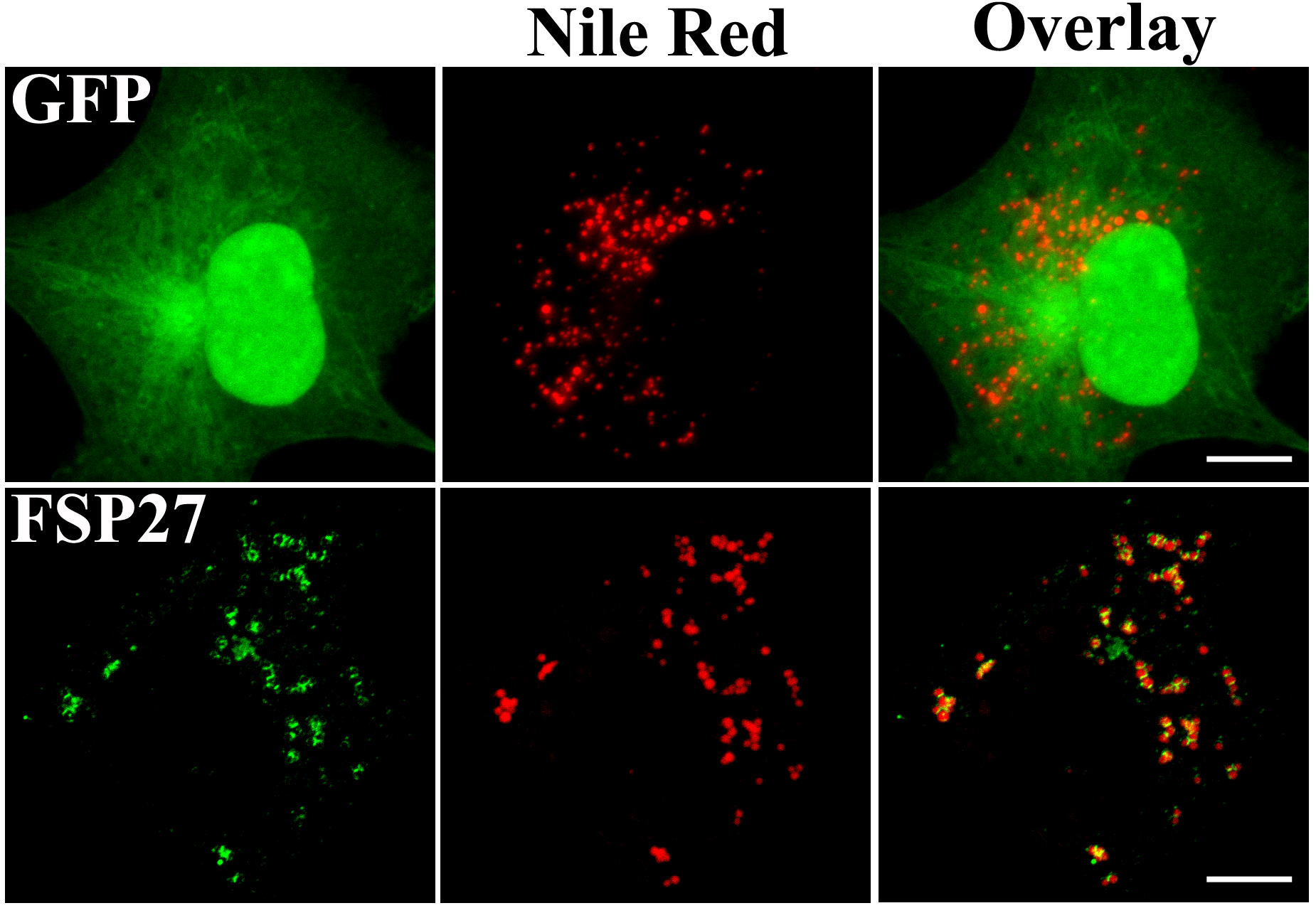

Supplement: Figure S4 — FSP27-GFP expression in brown preadipocytes causes clustering of LDs. Brown preadipocytes after 16 hr of transfection with GFP (top panels) and FSP27-GFP (bottom panels). LDs were stained with Nile red (middle panel). (TIF) [file pone.0028614.s004.tif]

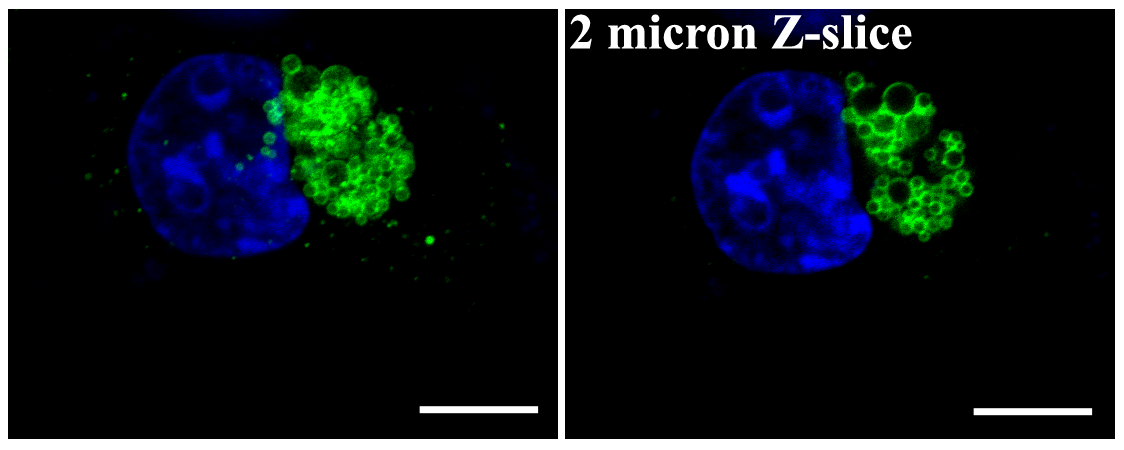

Supplement: Figure S5 — FSP27-GFP causes clustering of LDs in differentiating 3T3-L1 adipocytes. FSP27-GFP was expressed in differentiating 3T3-L1 adipocytes on day 3. 16 hr after transfection the cells were fixed and observed under confocal microscope. Left panel shows the distribution of LDs where all the confocal Z-sections of the cell were stacked to form one single image. The right hand side panel shows a very thin slice of 2 µm showing clustered LDs. Bar, 10 µm. (TIF) [file pone.0028614.s005.tif]

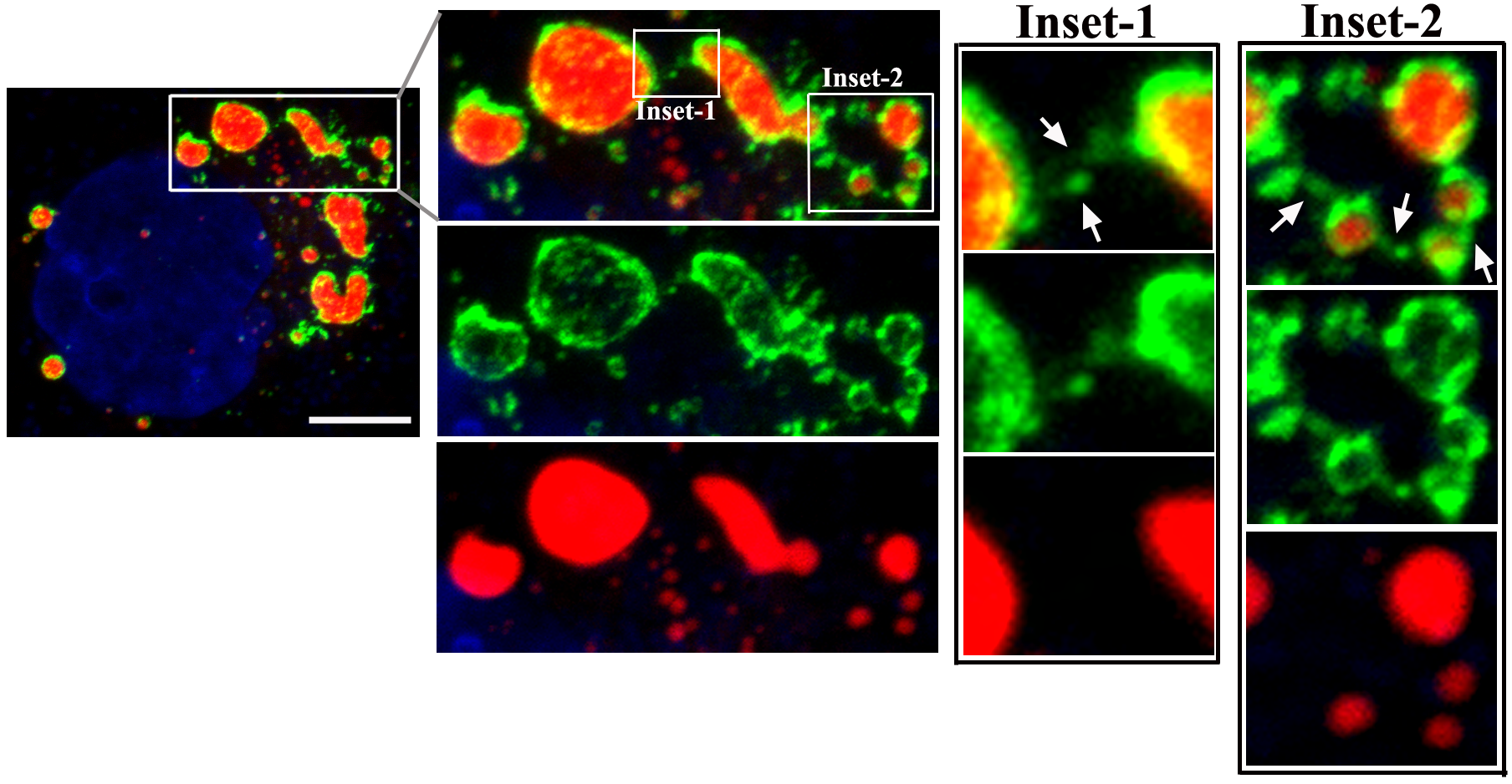

Supplement: Figure S6 — FSP27-GFP distribution during LD enlargement. FSP27 binds various LDs together by extending from one LD to another. The cells were transfected with FSP27-GFP, 4 hr after transfection cycloheximide was added for 1 hr and then the cells were fed with OA/BSA for 3 hr in the presence of cycloheximide. LDs were stained with Nile red. Inset shows that FSP27 is extended from one droplet to another (Arrows) and it is concentrated on most of the points from where it is extended. Bar, 10 µm. (TIF) [file pone.0028614.s006.tif]

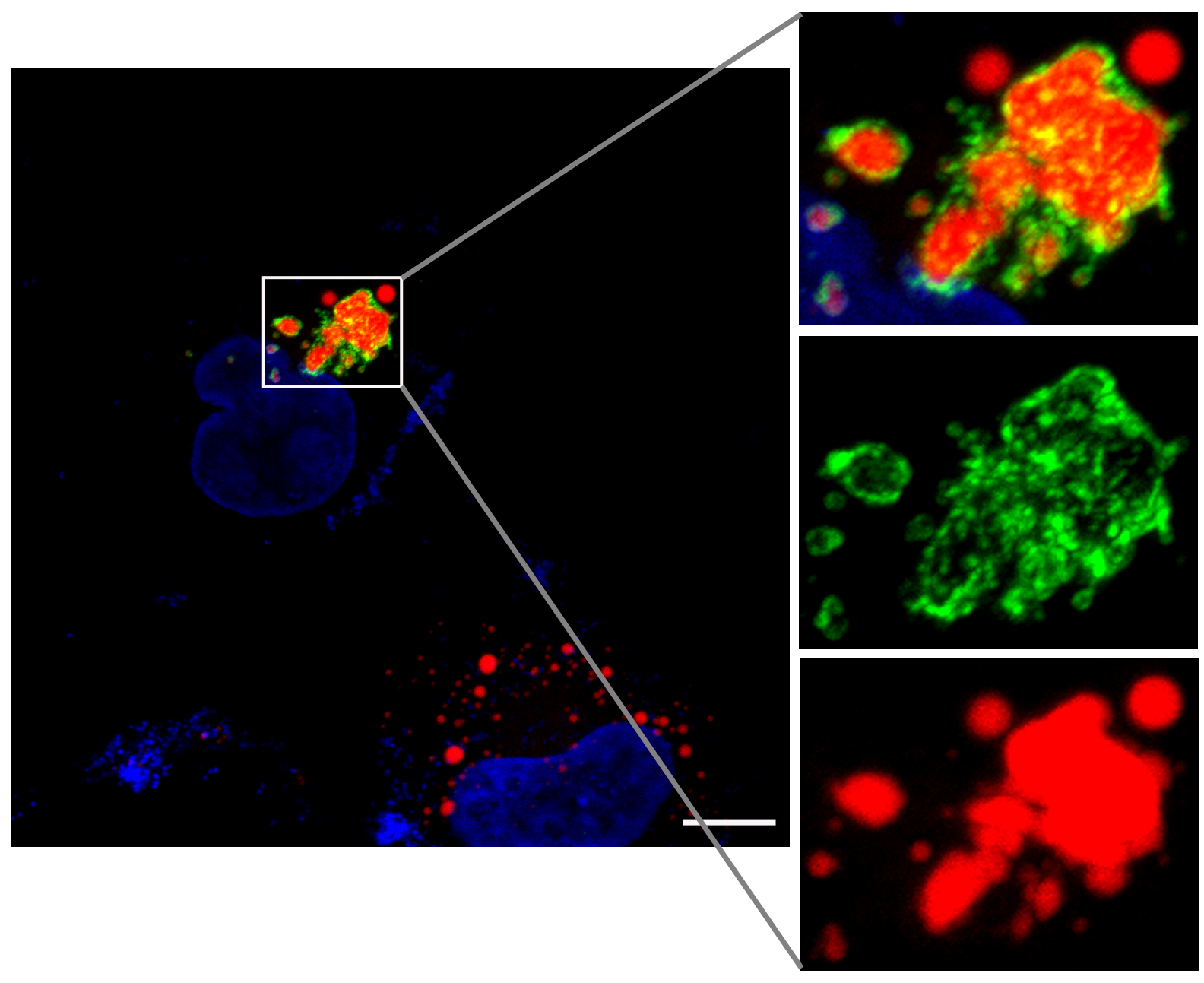

Supplement: Figure S7 — FSP27 might facilitate fusion of LDs. The cells were transfected with FSP27-GFP, 4 hr after transfection cycloheximide was added for 1 hr and then the cells were fed with OA/BSA overnight in the presence of cycloheximide. LDs were stained with Nile red. Note that FSP27 forms a kind of mesh around the droplets which gave an appearance as if they were coalescing. The untransfected cell within the same field has number of droplets distributed throughout the cytoplasm. Bar 10 µm. (TIF) [file pone.0028614.s007.tif]
